# Clinical baseline factors predict response to natalizumab: their usefulness in patient selection

**DOI:** 10.1186/1471-2377-14-103

**Published:** 2014-05-12

**Authors:** Alice Laroni, Ilaria Gandoglia, Claudio Solaro, Giuseppe Ribizzi, Tiziana Tassinari, Matteo Pizzorno, Sergio Parodi, Giovanna Baldassarre, Maria Teresa Rilla, Simonetta Venturi, Elisabetta Capello, Maria Pia Sormani, Antonio Uccelli, Giovanni Luigi Mancardi

**Affiliations:** 1 Department of Neurosciences, Rehabilitation, Ophthalmology, Genetics, Maternal and Child Health, University of Genova, Largo Daneo 3, 16132 Genova, Italy; 2 Neurology Unit, PA Micone Hospital Dip. Testa-Collo, ASL 3 Genovese, Genova, Italy; 3 Multiple Sclerosis Center, San Martino Hospital, Genova, Italy; 4 Multiple Sclerosis Center, Santa Corona Hospital, Pietra Ligure, Italy; 5 Division of Neurology, San Paolo Hospital, Savona, Italy; 6 Multiple Sclerosis Center, Sant’Andrea Hospital, La Spezia, Italy; 7 Multiple Sclerosis Center, Giovanni Borea Hospital, Sanremo, Italy; 8 Multiple Sclerosis Center, Imperia Hospital, Imperia, Italy; 9 Multiple Sclerosis Center, Galliera Hospital, Genova, Italy; 10 Department of Health Sciences, University of Genova, Genova, Italy

**Keywords:** Multiple sclerosis, Natalizumab, Neuropharmacology, Clinical neurology

## Abstract

**Background:**

Optimal patient selection would improve the risk-benefit ratio of natalizumab treatment for relapsing-remitting multiple sclerosis (RR MS). Clinical features of subjects responding to natalizumab have not been univocally recognized.

**Methods:**

Longitudinal data on RR MS patients treated with natalizumab in Liguria, Italy are reported. Predictors of relapse occurrence and disability improvement were analyzed with a logistic regression method in subjects treated for one year (N = 62). A new score, called “Better EDSS Trend (BET)”, was devised to describe the impact of the treatment on disability. Changes in annualized relapse rate (ARR) and Expanded Disability Status Scale (EDSS) after one and two years and proportion of disease-free patients were evaluated.

**Results:**

Previous EDSS worsening plus ARR ≥ 2 increased the risk of relapse during the treatment [Odds Ratio (OR) 4.12, P = 0.04], but this was not associated with an increase in disability at one year. EDSS 3.0-3.5 or high disease activity were associated with neurological improvement in the first year of treatment (respectively OR 5.78, P = 0.05 and OR 4.80, P = 0.05). Positive BET score, i.e. improvement in the disability trend, was observed in 40.3% of patients, and correlated with high ARR in the year before treatment (OR 1.69, P = 0.03).

**Conclusion:**

Subjects with EDSS 3.0-3.5 and those with very active disease in the year before treatment are most likely to improve in neurological function under natalizumab. A relapse in the first year of treatment is associated to high pre-treatment disease activity; however, since the occurrence of a relapse did not have a negative impact on clinical improvement at one year, we suggest that it should not lead to treatment discontinuation. We propose BET as an additional endpoint of treatment response in MS.

## Background

The marketing of natalizumab (Tysabri, Biogen Idec) for the treatment of relapsing remitting multiple sclerosis (RR MS) has been one of the major changes in the treatment of MS in recent years, due to its high efficacy [1]. However, the risk of the severe, sometimes fatal, disease called progressive multifocal leukoencephalopathy (PML), in about 2:1000 treated patients raises concerns [2,3]. Optimal pre-treatment patient selection is therefore required to minimize the risk-benefit ratio of such treatment. While longitudinal cohorts of patients treated with natalizumab have been published so far, results could be biased by issues related to the characteristics of the natural history of MS before and after initiation of treatment.

Due to the fluctuating course of MS, methodological pitfalls may affect the analysis of changes in relapse rate. Published studies on natalizumab effect report a marked decrease in mean relapse rate in treated patients, compared to pre-treatment (see for instance [4-7]). However, such phenomenon could be, at least partially, explained by the regression to the mean phenomenon, which is observed in patients with high relapse rate (such as in patients selected for treatment with natalizumab in European countries), as shown in placebo arms of clinical trials in MS [8]; alternatively, transition to a secondary progressive (SP) course might cause a decrease in relapse rate over time [9]. Moreover, descriptors used to characterize the clinical response to natalizumab, like the change in disability scores over time, appear sometimes inadequate because they do not include an analysis of the pre-treatment period.

The objective of this study was to analyze and describe clinical characteristics that might reflect a positive response to natalizumab in a cohort of MS patients, to test a new parameter for evaluating disability changes over time in MS patients and to provide clinicians and patients with baseline factors that could influence the treatment outcome, with a critical attention to methodological pitfalls.

## Methods

**Figure 1:**
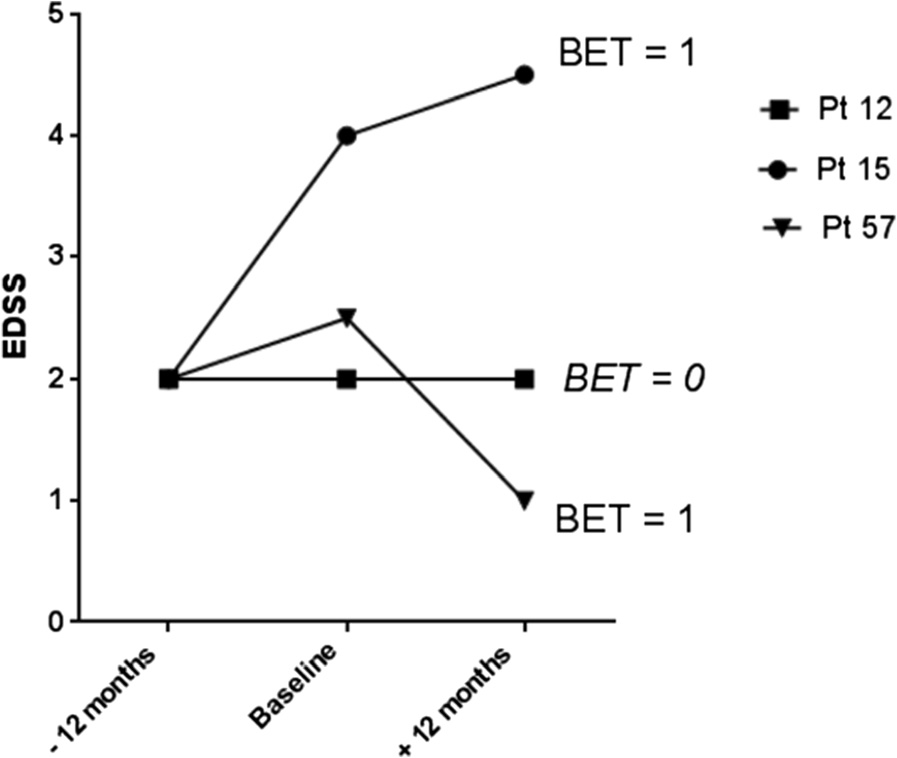
**Clinical course before and during natalizumab of three subjects is displayed.** Pt = patient. BET is = 1 in the case of both patients 15 and 57: Patient 15 worsened by 2 EDSS points in the year prior to treatment, while in the year following treatment he/she worsened by less than one EDSS point. Patient 57 worsened by 0.5 EDSS point in the year prior to treatment, while in the year following treatment he/she improved by 1.5 EDSS point. In both cases, patients experienced a decrease by at least one point in the extent of worsening in EDSS during the year of treatment as compared to that in the year prior to treatment. In contrast, Patient 12 had stable disability throughout the before and after treatment periods.

The study is composed of two parts. In the first part, we analyzed the characteristics of the clinical response to natalizumab in the first two years of treatment; in the second part, we looked for baseline parameters that would predict treatment response in the first year. The cohort was composed of all patients with relapsing MS treated monthly with natalizumab (300 mg by intravenous infusion) in the Liguria region, North-West of Italy, for at least one year by November 11, 2010 (N = 62). These patients had been followed for at least one year prior to treatment. An additional six patients had been enrolled in the cohort but interrupted the treatment at various times before the end of the first year, due to allergic reactions in four patients, “natalizumab inefficacy” in one patient and presence of anti-natalizumab antibodies in the remaining patient. A “worst scenario” additional analysis was performed assuming that these patients had been followed for one year of treatment with a negative outcome. Demographics, disease history, and clinical data of the 62 patients for the year prior to treatment were recorded at baseline (Table 1). Follow-up data were prospectively acquired at 6, 12, and 24 months after the start of treatment, and included disability, measured with the Expanded Disability Status Scale (EDSS), number of relapses, and side effects. At the time of the analysis, 30 of the 62 MS patients studied had been treated for at least 12 months and the remainder 32 for at least 24 months.For the description of clinical response to natalizumab, we analyzed the changes in annualized relapse-rate (ARR) and EDSS for the first and second year of treatment and the proportion of patients with relapses or at least 1-point of EDSS change compared to baseline. EDSS scores were acquired in relapse-free phase. We defined “clinically disease free” the subjects without relapses or 1-point EDSS increase during the first year of therapy. Improvement or stabilization of EDSS after treatment, compared to baseline EDSS, are usually evaluated as markers of response to the therapy. However, such measures do not consider the EDSS trend in the pre-treatment period and, therefore, may overestimate the effect of treatment on disability. Hence, we devised a new qualitative score as a tool to describe the effect of the treatment in decreasing the disability gain compared to the pre-treatment. In detail, we recorded the EDSS score 12 months before the start of treatment (“EDSS T-12”), at the start of treatment (“EDSS T0”), and 12 months after the start of treatment (“EDSS T + 12”) (N = 53 patients). We defined “Better EDSS trend” (BET) score a qualitative index of the trend in disability course from one year pre-treatment to one year after treatment. BET score values would be “1” in case of a decrease by at least one point in the extent of worsening in EDSS during the year of treatment as compared to that in the year prior to treatment, and “0” otherwise. Thus, BET = 1 is taken as (EDSS T0 - EDSS T-12) – (EDSS T + 12 - EDSS T0) ≥ 1. According to this definition, patients experiencing stability in the pre-treatment year and then throughout the first year of treatment have a BET score = 0. Figure 1 is given as an example.

**Table 1:** Baseline characteristics of the cohort

**Number of MS patients**	**62**
Sex	43 F, 19 M (69.4%–30.6%)
Age, mean (range)	37.5 years (20–60)
Disease duration, mean (range)	8.89 years (0–32)
ARR previous year (range)	2.26 (0–6)
ARR previous year, categorized	0-1 relapse: N = 21; 2 relapses, N = 22; ≥ 3 relapses, N = 19
Mean EDSS gain in the year before	+0.41 (SD 1.26)*
Mean baseline EDSS	4.1 (range 0–7.5)
Baseline EDSS, categorized	EDSS 0–2.5, N = 16; EDSS 3.0–3.5: N = 15, EDSS 4.0-5.5: N = 15, EDSS ≥6.0:, N = 16

*Known in 53/62.

In the second part of the study, we studied whether baseline parameters, including demographics and disease characteristics, were associated with the occurrence of relapses, disease freedom or BET score in the first year of treatment, using a logistic regression analysis.

Patients gave informed consent for data on their clinical history and follow-up to be used for the study. The study was approved by the Ethical Committee of San Martino University Hospital, Genova.

### Statistical analysis

Statistic tests included Wilcoxon matched-pairs signed rank test, Friedman test for multiple comparisons with Dunn’s post test, Mann–Whitney test, univariate and multivariate logistic regression. Data were analyzed with the SPSS (version 13.0) and GraphPad Prism (version 5) softwares. A P value < = 0.05 was considered significant.

## Results

### Relapse rate and disability before and after treatment

**Figure 2:**
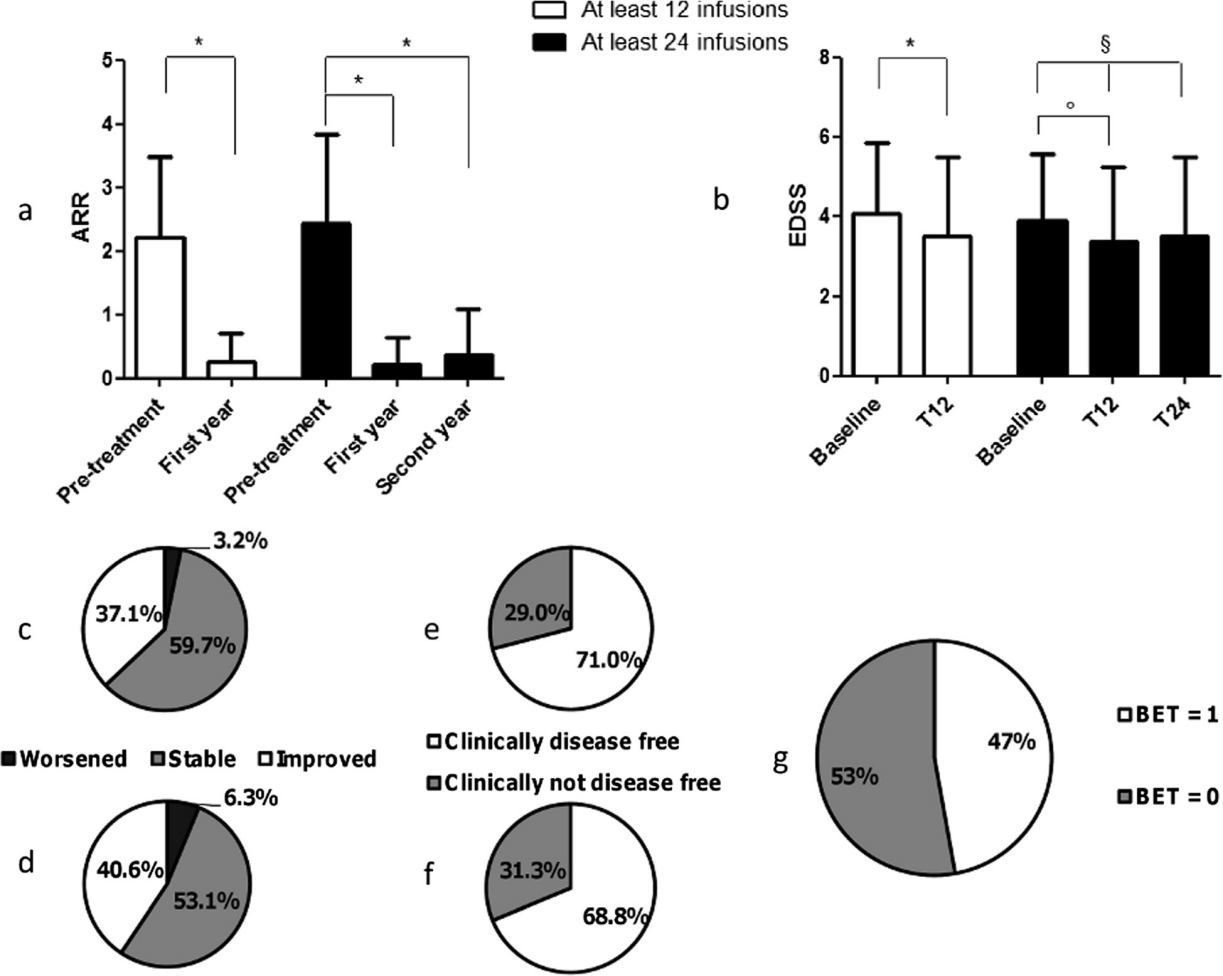
**Clinical effect of natalizumab in patients treated with 12 or 24 monthly infusions. a)** Changes in annualized relapse rate among the pre-treatment year and the follow-up, *P < 0.0001; **b)** Changes in mean EDSS over time *P < 0.0001, ^§^P = 0.009 (Friedman test), °P < 0.05 (Dunn’s post test); **c)** Percentage of patients with improvement by at least 1 point, worsening by at least one point, or stable EDSS score in the first year of treatment compared to baseline and **d)** in the second year; **e)** Percentage of disease-free patients in the first year and **f)** in the second year of treatment; **g)** percentage of patients with BET = 1 within the first year of treatment.

Both mean relapse rate and mean disability decreased significantly in the first year of treatment and remained stable in the second year. Mean ARR decreased from 2.26 in the year prior treatment to 0.26 in the first year of treatment (P < 0.0001) in patients treated with at least 12 monthly infusions, and from 2.44 to 0.22 (first year) and 0.38 (second year) in patients treated for two years (P < 0.0001) (Figure 2a). About one fourth of the subjects (16/62 subjects, 25.8%) had a relapse in the first year of treatment and 9/32 (28.1%) in the second year of treatment. Mean EDSS decreased from 4.1 to 3.6 at the six-month follow-up and 3.5 at the 12-month follow-up (P < 0.0001 among baseline and 12-month follow up). A similar trend was observed in patients treated for two years and whose mean disability remained stable in second year of treatment, with the EDSS changing from 3.9 (baseline) to 3.4 (first year) and 3.5 (second year) (Friedman test P = 0.009 with Dunn’s post test showing significant difference among baseline and first year) (Figure 2b). Though clinical data, and particularly disability scores, were acquired in relapse-free phases, we cannot exclude that baseline EDSS scores may be influenced, in some cases, by recent relapses, since natalizumab is prescribed to patients with active disease in the pre-treatment.After one year of treatment, 23/62 (37.1%) patients had a decrease of EDSS by at least one point compared to baseline while 2/62 (3.2%) had an increase of EDSS by at least one point (Figure 2c). After two years, 13/32 (40.6%) had a decreased EDSS score compared to baseline while 2/32 (6.25%) had worsened (Figure 2d). Freedom from clinical disease occurred in 44/62 (71%) patients in the first year (Figure 2e) and in 22/32 (68.8%) patients in the second year (Figure 2f).

About 50% of patients in the first year of treatment had BET score = 1 (25/53, 47.2%) (Figure 2g) and this was independent on the occurrence of a relapse during the same time-period (data not shown). Although there was a strong association between EDSS improvement and BET = 1 (χ^2^ test, P < 0.0001), eight patients had BET = 1 in the absence of EDSS improvement. Of these, 5 patients had been worsening in the pre-treatment and were stable during the treatment, while the other 3 had a less pronounced worsening of disability as compared to the pre-treatment.

### Predictors of treatment response

A. Relapses

**Figure 3:**
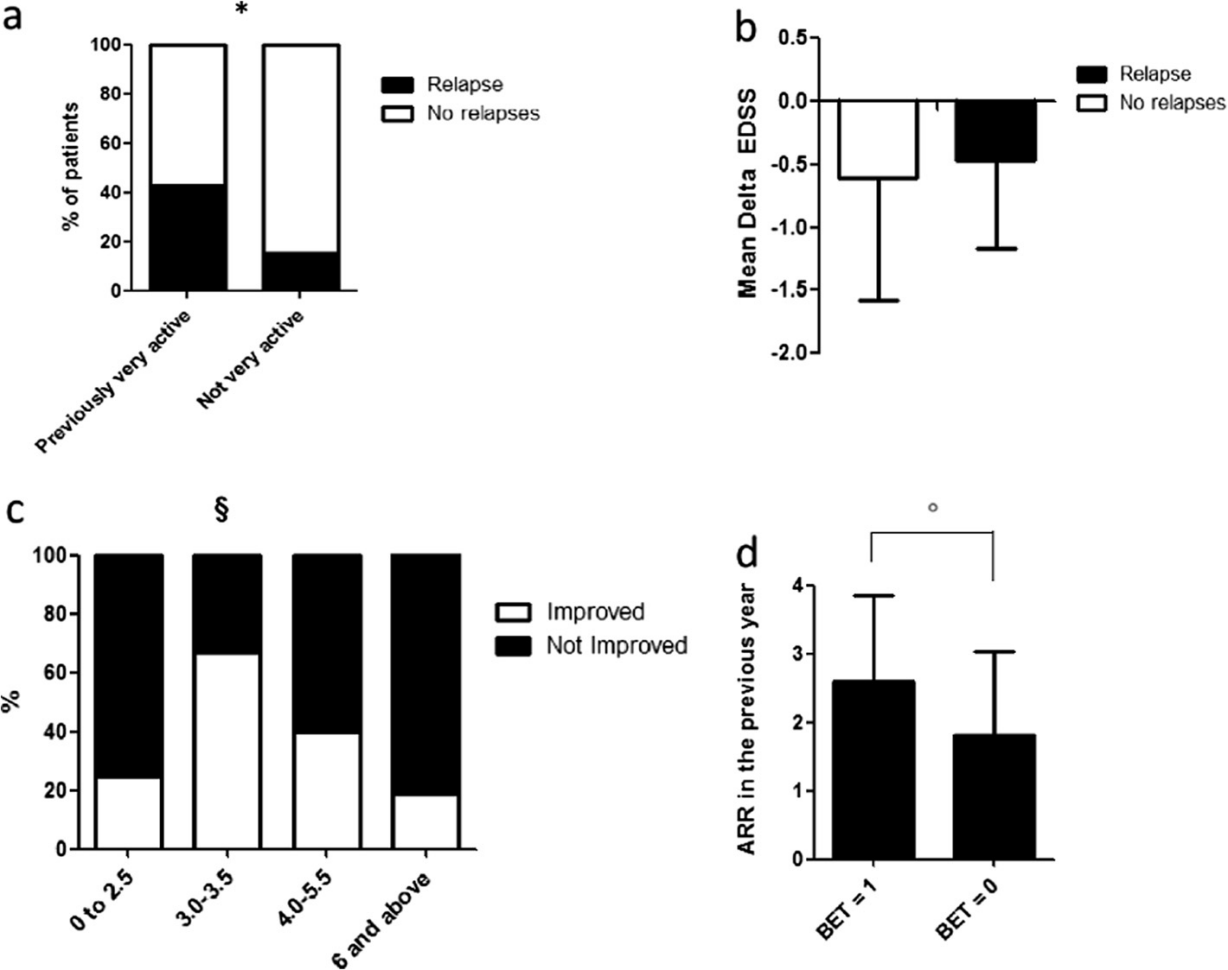
**Baseline predictors of response to natalizumab. a)** A relapse in the first year was more frequent in very active patients, i.e. patients with at least 2 relapses and ≥ 1 EDSS gain in the year before starting natalizumab, *P = 0.04 **b)** Improvement in EDSS score after one year of treatment was not different in patients who had or had not experienced a relapse during that time; **c)** 1-point EDSS improvement was more frequent in patients with baseline EDSS 3.0-3.5 (^§^P = 0.02, univariate logistic regression); **d)** Patients with BET score = 1 had significantly higher pre-treatment relapse-rate compared to those with BET score = 0 (°P = 0.011).

We were able to predict the occurrence of a relapse in the first year of treatment. Specifically, we found that patients who had experienced more than one relapse and concomitant worsening of EDSS scale by at least one point in the year prior to treatment (i.e. “very active patients”) were more likely to relapse during the treatment (Odds Ratio 4.12, 95% Confidence interval, 1.05 to 16.23 P = 0.04) (Table 2 and Figure 3a).Relapses in the first year of treatment did not lead to an increase in disability or a lesser clinical improvement (Figure 3b).

**Table 2:** Predictors of occurrence of relapse in the first year of treatment

	**Univariate analysis**				**Multivariate analysis**	
	**OR**	**CI**	**P**	**OR**	**CI**	**P**
Sex (female vs male)	2.31	0.57–9.32	0.24	-	-	-
Age	1.00	0.94–1.07	0.88	-	-	-
Disease duration	1.01	0.92–1.11	0.81	-	-	-
ARR in the pre-treatment year	0.98	0.63–1.54	0.93	-	-	-
EDSS gain the pre-treatment year	1.53	0.89–2.62	0.12	-	-	-
EDSS at baseline	1.25	0.90–1.75	0.18	-	-	-
*EDSS at baseline, categorized*			0.62	*-*	*-*	*-*
3.0 to 3.5 compared to < = 2.5	0.08	0.18–6.44	0.93	-	-	-
4.0 to 5.5 compared to < = 2.5	1.58	0.29–8.61	0.6	-	-	-
6 and above compared to < = 2.5	2.60	0.52–13.04	0.24	-	-	-
**High pre-treatment disease activity**	**4.12**	**1.05–16.23**	**0.04**	**4.12**	**1.05–16.23**	**0.04**

OR: odds ratio. CI = 95% Confidence Interval.

Univariate and multivariate logistic regression analysis.

No other single baseline parameter, including sex, age, disease duration, ARR in the year prior to treatment, EDSS, or EDSS gain in the year prior to treatment were associated with the occurrence of a relapse in the first year of therapy. “Worst scenario” univariate and multivariate logistic regression analyses, including the six patients who dropped out before reaching one year of treatment and assuming those patients having a relapse during the first year, did not show an association between high pre-treatment disease activity and occurrence of relapses in the first year.

B. Disability

High pre-treatment disease activity was significantly associated with a 1-point EDSS improvement at the one-year follow-up (Odds Ratio 4.58, 95% Confidence Interval 1.25-16.76, P = 0.02), which was also predicted by baseline EDSS included between 3.0 and 3.5 (Odds Ratio 6.00, 95% Confidence interval 1.26-28.55, P = 0.02) (Table 3 and Figure 3c). EDSS improvement was not associated with sex, age, pre-treatment relapse rate, EDSS worsening in the pre-treatment year or disease duration. The association between high pre-treatment disease activity and EDSS improvement was confirmed by a “worst scenario” analysis including the six patients who dropped out before one year, assuming they would not have an improvement of EDSS under treatment.

**Table 3:** Predictors of 1-point EDSS improvement in the first year of treatment

	**Univariate analysis**				**Multivariate analysis**	
	**OR**	**CI**	**P**	**OR**	**CI**	**P**
Sex (female vs male)	1.02	0.33–3.11	0.98	-	-	-
Age	0.1	0.94–1.51	0.91	-	-	-
Disease duration	1.00	0.92–1.09	0.91	-	-	-
ARR in the pre-treatment year	1.3	0.96–1.95	0.21	-	-	-
EDSS gain the pre-treatment year	1.31	0.83–2.07	0.25	-	-	-
EDSS at baseline	0.91	0.68–1.22	0.54	-	-	-
*EDSS at baseline, categorized*			**0.04**				**0.056**
** 3.0 to 3.5 compared to < = 2.5**	**6.00**	**1.26–28.55**	**0.02**	**5.78**	**1.06–31.50**	**0.04**	
4.0 to 5.5 compared to < = 2.5	2.00	0.43–9.26	0.37	0.73	0.11–4.8	0.74	
6 and above compared to < = 2.5	0.69	0.13–3.75	0.67	0.59	0.08–4.2	0.59	
**High pre-treatment disease activity**	**4.58**	**1.25–16.76**	**0.02**	**4.80**	**1.00–23.08**	**0.05**	

OR: odds ratio. CI = 95% Confidence Interval.

Univariate and multivariate logistic regression analysis.

None of the baseline parameters predicted freedom from clinical disease in the first year (data not shown).

Higher ARR in the year prior to treatment increased the possibility of BET score = 1(Odds Ratio 1.69, 95% Confidence interval 1.04-2.75, P = 0.03; Mean ARR in patients with BET = 1: 2.60 ± 1.3 vs 1.52 ± 1.2, Mann–Whitney test P = 0.011) (Table 4 and Figure 3d); no association was found with other baseline parameters like baseline EDSS, sex, age, disease duration or with baseline EDSS class. Due to the formula used to calculate BET, which included pre-and post-treatment disability changes, EDSS worsening in the pre-treatment year was not considered among analyzed baseline parameters. Results were confirmed by a “worst scenario” analysis where the six dropped-out patients were assumed to have BET score = 0 after one year of treatment.

**Table 4:** Predictors of BET score = 1 in the first year of treatment

	**Univariate analysis**				**Multivariate analysis**	
	**OR**	**CI**	**P**	**OR**	**CI**	**P**
Sex (female vs male)	1.18	0.38–3.70	0.78	-	-	-
Age	1.02	0.96–1.09	0.46	-	-	-
Disease duration	0.98	0.89–1.07	0.63	-	-	-
**ARR in the pre-treatment year**	**1.69**	**1.04–2.75**	**0.03**	**1.69**	**1.04–2.75**	**0.03**
EDSS gain the pre-treatment year	*n.a.*^‡^	*n.a.*	*n.a.*	*n.a.*	*n.a.*	*n.a.*
EDSS at baseline	0.91	0.67–1.25	0.58	-	-	-
*EDSS at baseline, categorized*			0.19	-	-	-
3.0 to 3.5 compared to < = 2.5	2.00	0.46–8.78	0.36	-	-	-
4.0 to 5.5 compared to < = 2.5	3.00	0.62–14.62	0.17	-	-	-
6 and above compared to < = 2.5	0.50	0.09–2.64	0.41	-	-	-
High pre-treatment disease activity	*n.a.*	*n.a.*	*n.a.*	*n.a.*	*n.a.*	*n.a.*

^‡^: n.a.: not applicable. OR: odds ratio. CI = 95% Confidence Interval.

Univariate and multivariate logistic regression analysis.

### Side effects

Of the 62 patients analyzed, 40 reported side effects that did not warrant discontinuation of the treatment, of which the most frequent were infections (33/62 patients), fatigue (20/62 patients), headache (12/62 patients), and musculoskeletal pain (9/62 subjects). One subject had a recurrence of depressive bipolar disorder and attempted suicide, recovering well after hospitalization and a change in her anti-psychotic treatment. Two patients were diagnosed with pityriasis rosea.

## Discussion

In this study, we followed a cohort of MS patients treated with natalizumab for one to two years, with the aim of identifying the best markers of response to the treatment and defining the characteristics of responders. Most published studies on this topic are descriptive [5,7,10-16]. Few research papers have attempted to correlate baseline data with outcome towards prediction of treatment response, and results are not always consistent between studies, possibly due to the methodological biases that might affect uncontrolled longitudinal studies. Among predictors of decreased relapse rate, Fernandez et al. report baseline EDSS lower than 6 [17], while according to another study, which failed to find an association with baseline disability categories (similar ARR under natalizumab in subjects with EDSS 0–3.5 and subjects with EDSS 3.5–6.5), patients with shorter disease course have the lowest relapse rate under natalizumab [18]. Sargento-Freitas and co-authors found that patients with “optimal response” to natalizumab (those with sustained, relapse-independent reduction in EDSS score of at least 1 point or a reduction in ARR of more than 1 during NTZ treatment) had higher relapse-rate in the pre-treatment year compared to those with suboptimal or no response to treatment [19]. Prosperini et al. report that patients with fewer pre-treatment relapses or lower EDSS are more likely to be disease-free after two years of treatment [20].

In the cohort we report here, as well as in others published so far [4-7], mean ARR decreased to a very low value after natalizumab (0.26/year). As a consequence, the magnitude of changes in the ARR were directly proportional to the pre-treatment disease activity (the higher the relapse rate in the year prior to treatment, the greater the improvement). This might lead to misleading conclusions, such as considering natalizumab less effective in subjects with lower pre-treatment relapse rate (and higher EDSS). Therefore, we focused our analysis on predictors of relapse occurrence. About one-fourth of subjects had a relapse in the first year of treatment. We found that a high pre-treatment disease activity, as defined by at least two relapses and concomitant 1-point EDSS gain, increased the risk of a relapse in the first year of therapy, which was independent from baseline disability. Importantly, the patient population with a relapse under natalizumab had a similar mean decrease in EDSS after one year compared to those without relapse: therefore, relapses upon treatment did not lead to permanent EDSS worsening. If these data will be confirmed in a longer follow-up, this would differentiate natalizumab from interferon-beta, since relapses under treatment with interferon beta predict disability accumulation in the long-term [21]. We did not find predictors of disease freedom under treatment, differently from what reported by others [20], possibly due to the different definition of freedom from disease activity employed in our study (not including magnetic resonance imaging data).

High pre-treatment disease activity, as defined above, was associated to 1-point EDSS decrease after treatment, similar to what has been reported by the work by Belachew and co-authors [22]. While the same authors did not find associations between baseline disability and EDSS improvement, we found that baseline mild-moderate disability (EDSS 3.0-3.5) was a predictor of EDSS decrease under treatment; different ways of categorizing disability classes may explain this.

From the patient’s point of view, the decrease in disability is not the only relevant endpoint. Stabilization of disease, as well as slower progression, could significantly improve the quality of life. This observation prompted us to create a novel outcome, which we called BET, that describes an improved EDSS trend in the first year under treatment, compared to the year prior to treatment. We found that about half treated patients had a BET score = 1 (improved EDSS trend) after one year of treatment with natalizumab. We show that BET score = 1 correlated with relapse rate before treatment, indicating again that subjects with active disease in the pre-treatment were best responders. Baseline disability did not influence the BET score. It is important to note that, since the BET score describes the EDSS trend over time, it has some limitations associated to the EDSS scale (i.e. the non-linearity of the disability scale); at the same time, BET is easy to calculate in clinical practice, where EDSS is widely used. We suggest that BET score, which compares disability changes in two subsequent time frames, could be used as a tool for analyzing the impact of disease-modifying treatments in disability trends compared to the pre-treatment.

## Conclusions

In conclusion, we demonstrated that patients who would most likely improve their neurological status after a one-year treatment with natalizumab had a high disease activity in the year prior to treatment, i.e. at least two relapses and a 1-point increase in EDSS (independent of baseline disability), or a moderate disability (EDSS 3.0 to 3.5) at the time of natalizumab initiation. High pre-treatment disease activity increased the risk of a relapse during the therapy, but this did not impact upon the overall neurological improvement at one year after treatment. Therefore, we suggest that the occurrence of a relapse during natalizumab should not be considered as treatment failure. We propose BET score as an additional tool to describe the response to treatments in MS.

## Competing interests

AL received lecturing honoraria from Biogen-Dompé, Biogen Idec and Novartis and funding for travel from Bayer-Schering, Novartis, Biogen-Dompé, Biogen Idec, Merck Serono, and Sanofi Genzyme. IG reports received funding for travel from Biogen-Dompé and Biogen Idec. CS received lecturing honoraria from Biogen Idec, Merck Serono, TEVA, Almirall, Novartis and funding for travel from Bayer-Schering, Novartis, Biogen Idec, Merck Serono, Alimirall and Sanofi Genzyme. GR has no competing interests. TT received funding for travel from Lilly and Janssen. MP received funding for travel from Merck Serono. SP has no competing interests. GB received funding for travel from Biogen-Dompé. MTR has no competing interests. SV has no competing interests. EC received funding for travel by Biogen Dompé. MPS received funding for travel and consulting fee from Merck Serono, Biogen Idec, Actelion, Synthon and Allozyne. AU received financial support for research, honoraria for consultation, speaking or both at meeting for Genetech, Roche, Allergan, Merck-Serono, Sanofi-Aventis, Biogen-Dompé, Biogen Idec, Novartis. GLM has received honoraria for lecturing, funding for travel, and financial support for research from Bayer Schering, Biogen Idec, Sanofi Aventis, Teva, Merck Serono and Novartis Pharmaceuticals.

## Authors’ contributions

AL, MPS, AU and GLM conceived and designed the study. AL, IG, CS, GR, TT, MP, SP, GB, MTR, SV, EC, AU, GLM contributed to generation of the data. MPS and AL performed statistical analysis. AL, IG, GLM wrote the paper. All authors reviewed and approved the final manuscript.

## Pre-publication history

The pre-publication history for this paper can be accessed here:

http://www.biomedcentral.com/1471-2377/14/103/prepub
